# Quinine Bihydrochlorid in Scarlatina

**Published:** 1916-03

**Authors:** 


**Quinine Bihydrochlorid in Scarlatina.** Chichkine, Gac. Med. Catalan, Jan. 1915, uses sterile, 30% solutions hypodermatically, 1 C. C. for children of 1-2 years, 2 C. C. for those 3-4 years, and increasing 1 C. C. for each year thereafter (probably with a reasonable limit, as this dose is relatively high). A single injection is said to reduce the temperature rapidly and to improve the subsequent course.

